# Supplementary figures and images for: Extreme allelic heterogeneity at a Caenorhabditis elegans beta-tubulin locus explains natural resistance to benzimidazoles
Source: PLoS Pathog. 2018 Oct 29;14(10):e1007226. doi: 10.1371/journal.ppat.1007226 (PMC6224181; doi:10.1371/journal.ppat.1007226)

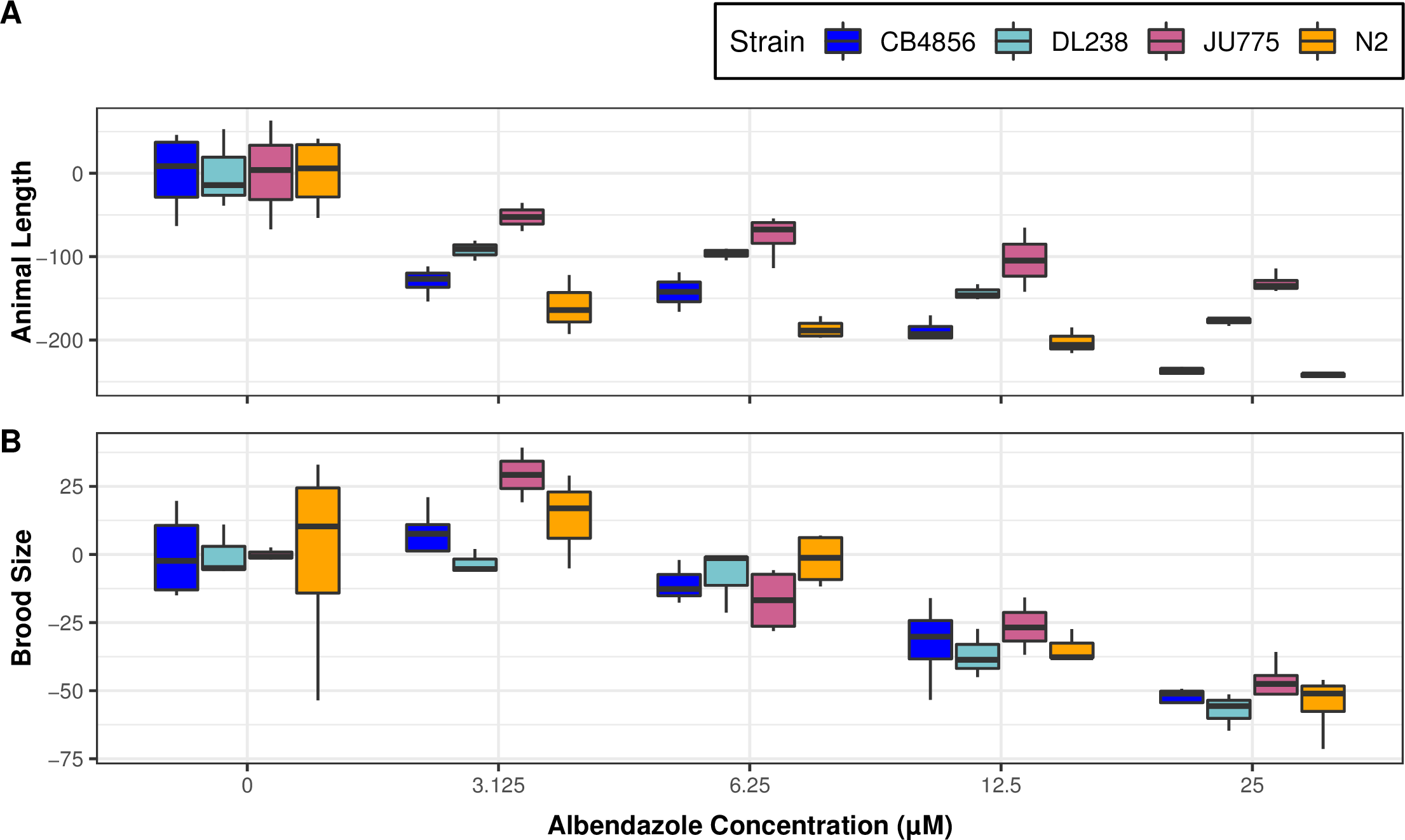

Supplement: S1 Fig — The box plots show a representative dose-response experiment on four C. elegans strains for (A) animal length (q90.TOF) and (B) brood size (norm.n). The ABZ concentration is plotted on the x-axis, and individual replicate trait values subtracted from the mean in DMSO control conditions is plotted on the y-axis. Each box represents four technical replicates. (TIF) [file ppat.1007226.s026.tif]

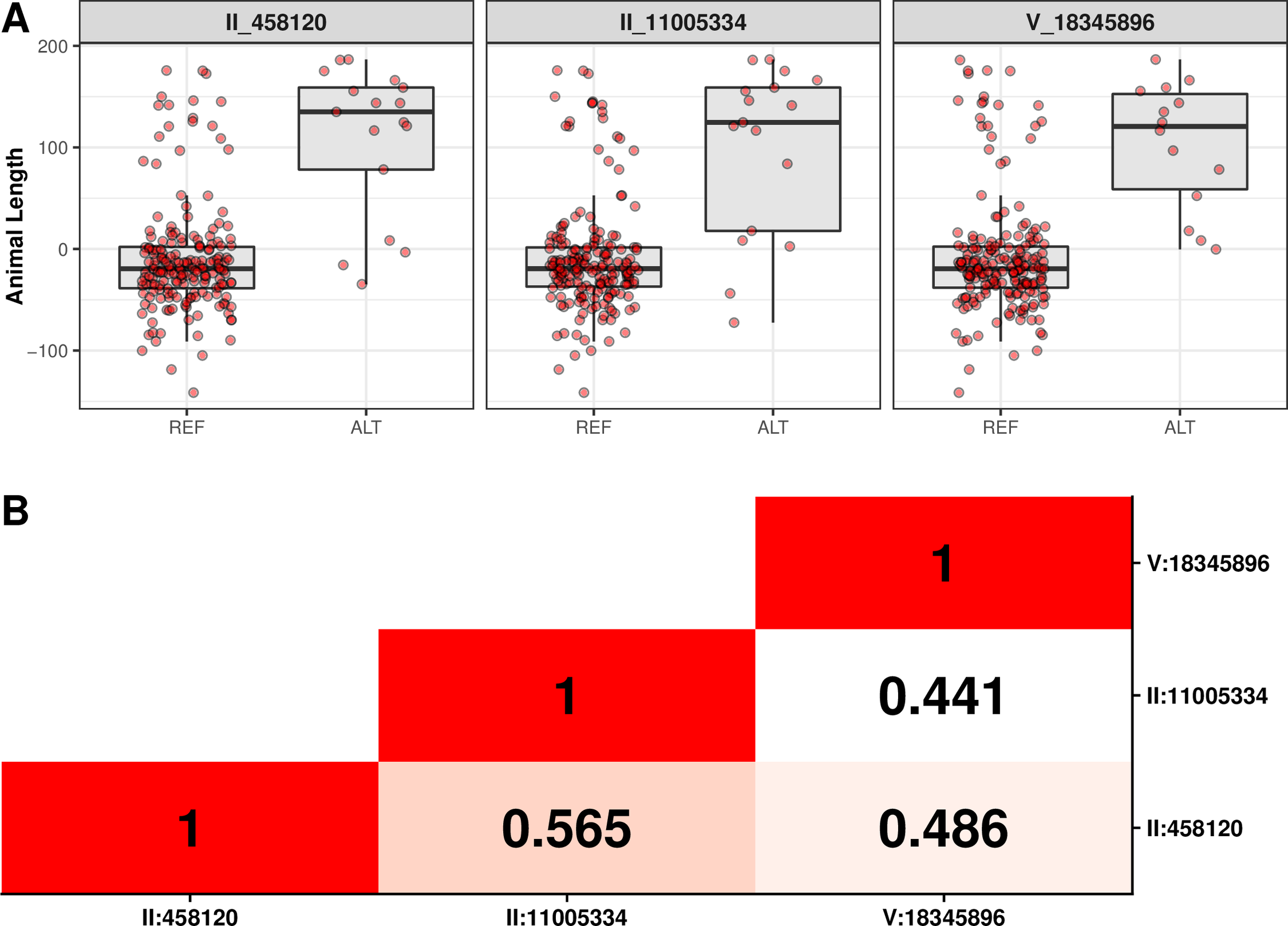

Supplement: S2 Fig — (A) Regressed animal length (q90.TOF) phenotypes in the presence of ABZ. Each dot represents the mean regressed animal length of four replicates. Strains are grouped by the presence of the REF or ALT genotype at the peak QTL marker identified in the single-marker GWA mapping approach. (B) Linkage disequilibrium (LD) as measured by the correlation coefficient between peak QTL markers in A. The formula for the correlation coefficient r = -D / sqrt (p(A) * p(a) * p(B) * p(b)). (TIF) [file ppat.1007226.s027.tif]

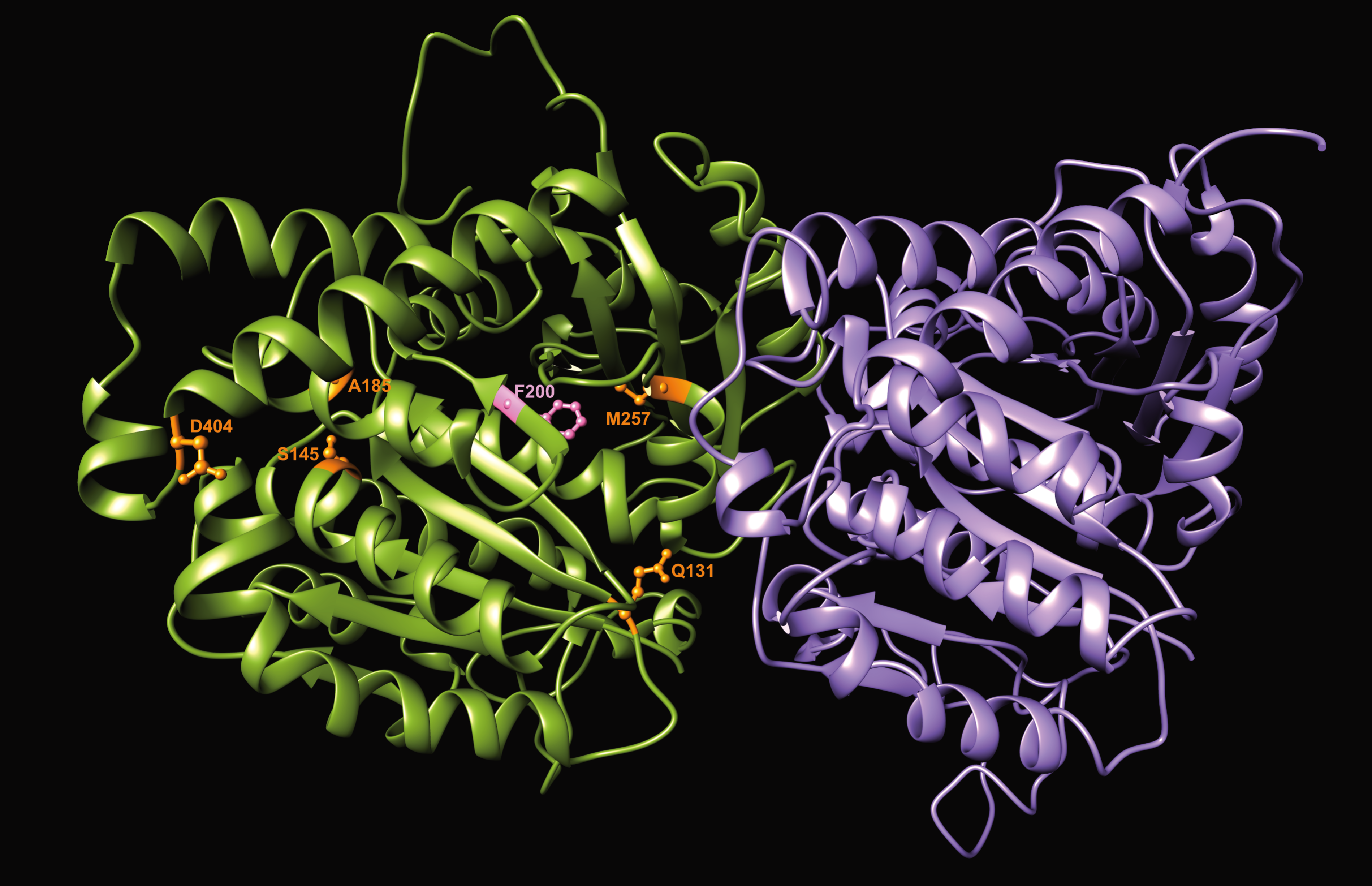

Supplement: S3 Fig — An in silico model of the BEN-1 structure (green), binding to an alpha-tubulin (purple) is shown. Novel identified amino acid substitutions among C. elegans wild strains are highlighted in color. Most alleles were correlated with ABZ resistance (orange). The F200Y mutation, known as a major BZ resistance marker in parasitic nematodes is shown in pink. (TIFF) [file ppat.1007226.s028.tiff]

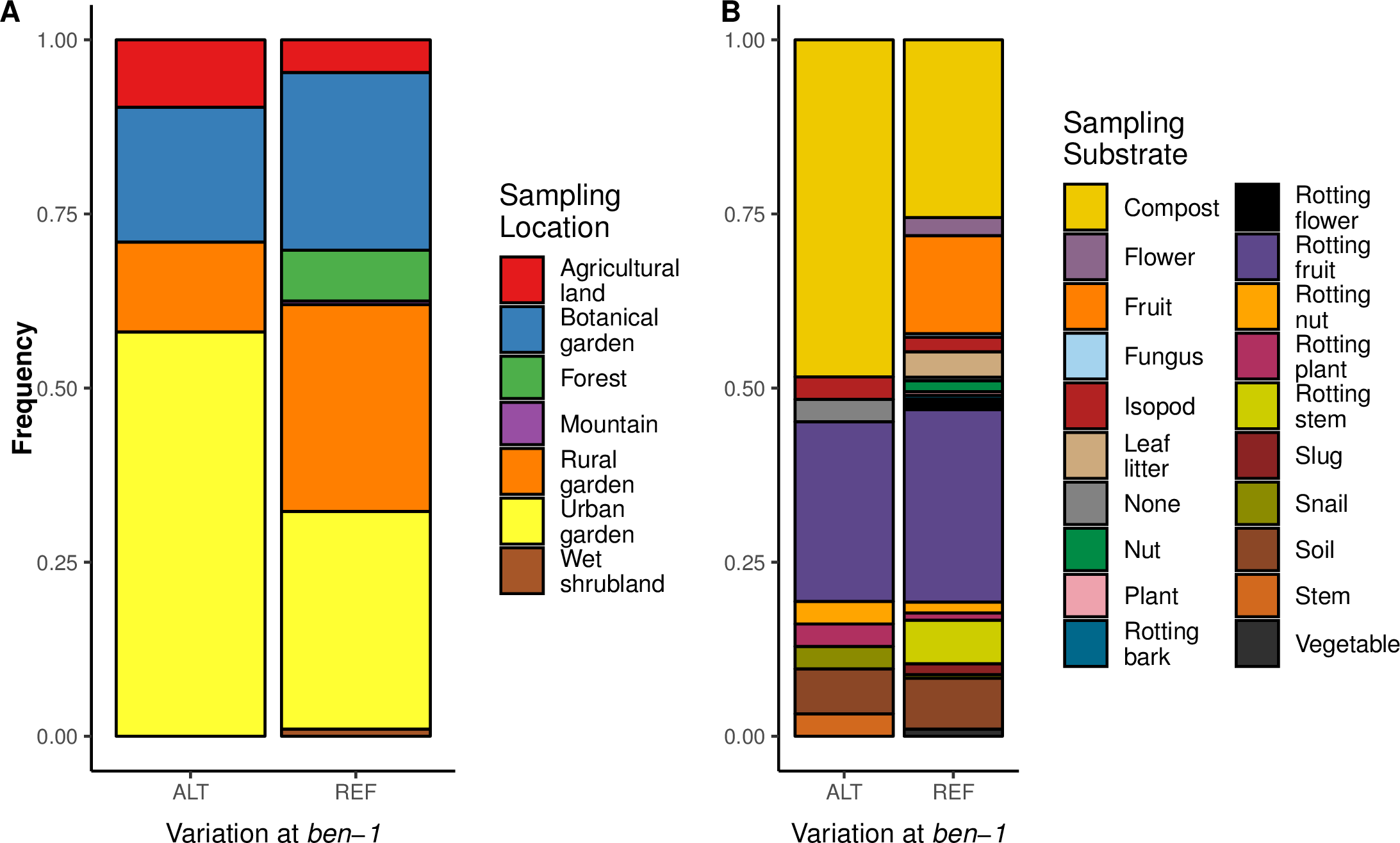

Supplement: S4 Fig — The fractions of wild C. elegans isolates sampled in a given (A) location and on a given (B) substrate are shown. Colors for the stacked bar plots correspond to different (A) sampling locations and (B) substrates. (TIF) [file ppat.1007226.s029.tif]

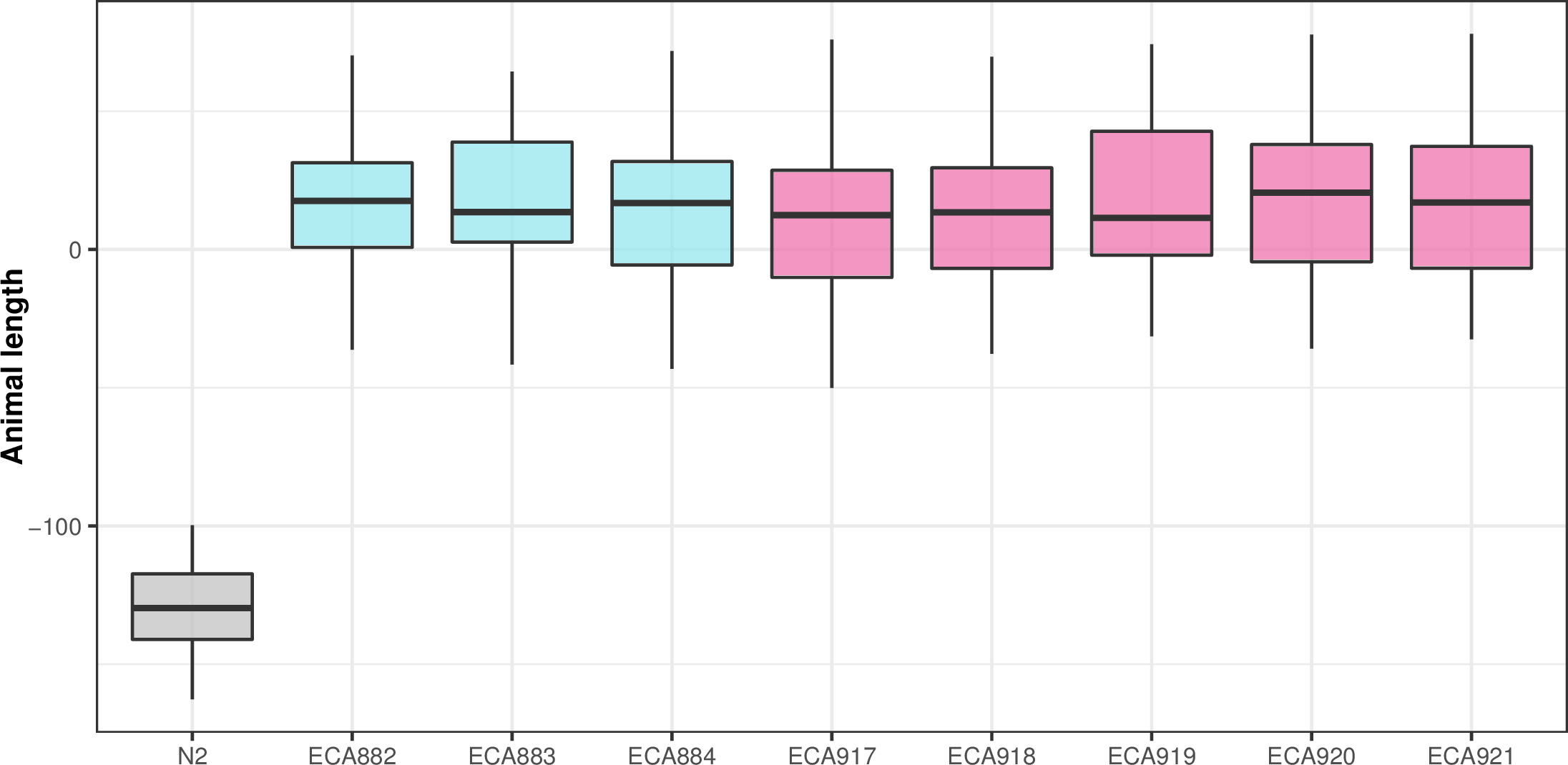

Supplement: S5 Fig — Tukey box plots of the animal length phenotypes of the generated ben-1 allele-replacement strains after ABZ treatment is shown. Blue boxes correspond to independent F200Y allele strains and pink boxes correspond to independent Del strains. The y-axis represents the animal-length phenotype after correcting for growth in DMSO conditions. (TIF) [file ppat.1007226.s030.tif]

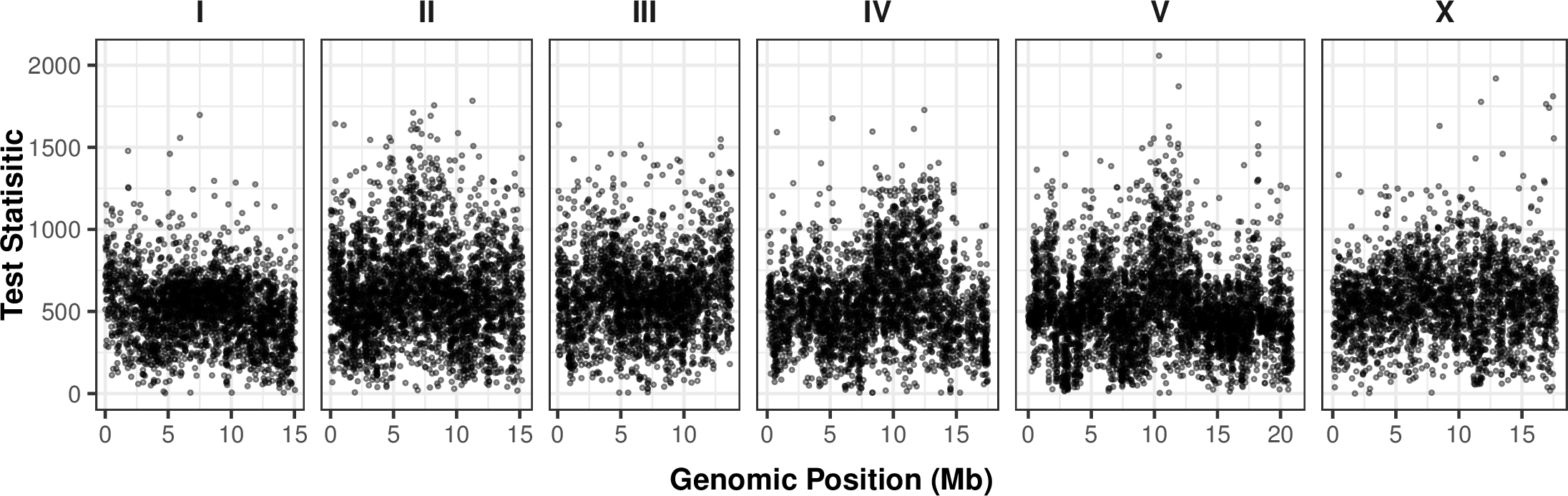

Supplement: S6 Fig — Gene-burden manhattan plot o the ben-1 corrected animal length (q90.TOF) after ABZ exposure. Animal-length phenotypes in the presence of ABZ were adjusted based on the presence of a putative loss-of-function variant in ben-1. Each dot represents a single gene of the C. elegans genome with its genomic location plotted on the x-axis, and the test statistic plotted on the y-axis. Genes passing the burden test statistical significance threshold are colored in red. (TIF) [file ppat.1007226.s031.tif]

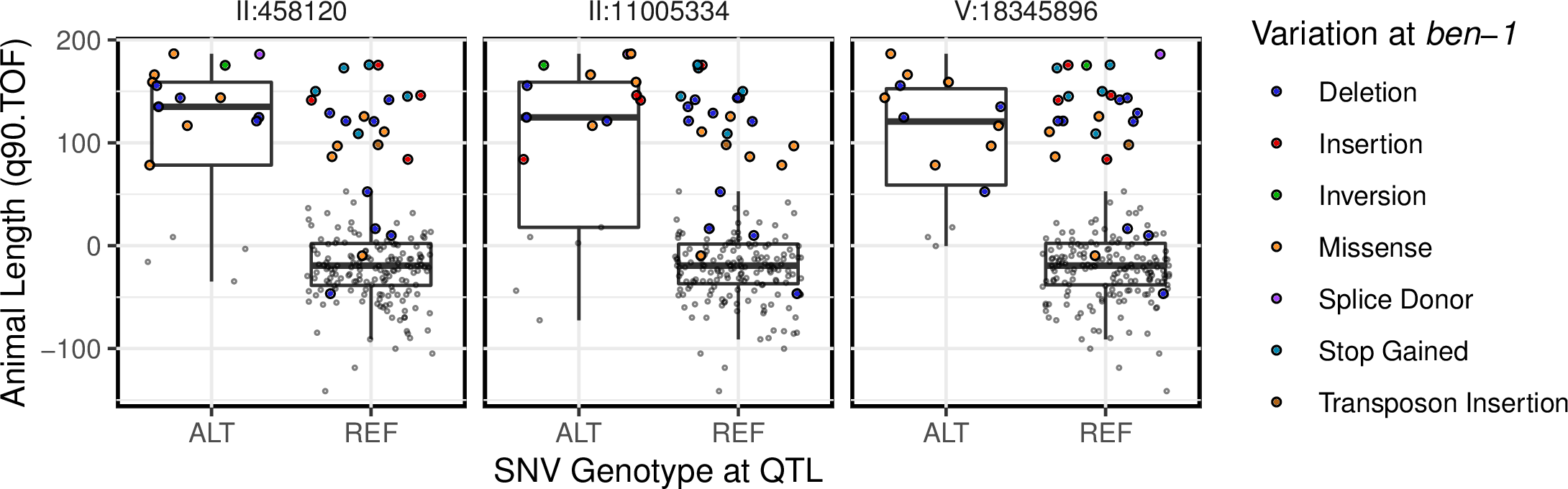

Supplement: S7 Fig — Regressed animal length (q90.TOF) phenotypes in the presence of ABZ. Each dot represents the mean regressed animal length of four replicates per strain. Strains are grouped by the presence of the REF or ALT genotype at the peak QTL marker identified in the single-marker GWA mapping approach. Dot colors correspond to the identified variant class in the ben-1 locus. (TIF) [file ppat.1007226.s032.tif]

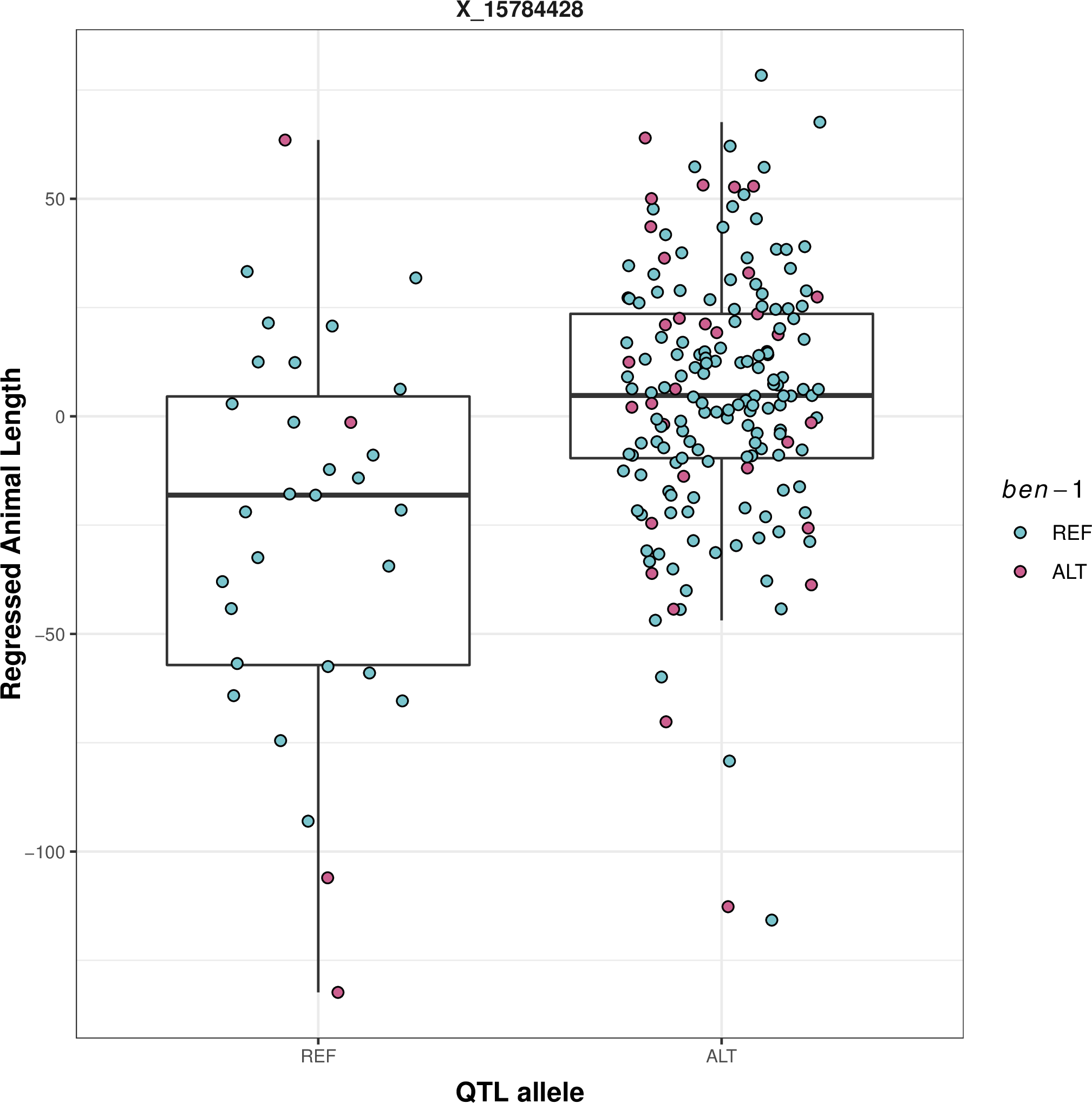

Supplement: S8 Fig — Animal length phenotypes in the presence of ABZ after correction for the presence of a putative loss-of-function variant in ben-1. Each dot represents the mean regressed animal length of four replicates per strain. Strains are split into groups based on the presence of the REF or ALT allele at the chromosome X QTL peak marker. Dots are colored by the presence of a putative loss-of-function variant in ben-1, where pink corresponds to strains with a putative loss-of-function variant, and blue corresponds to strains with no putative loss-of-function variants. (TIF) [file ppat.1007226.s033.tif]

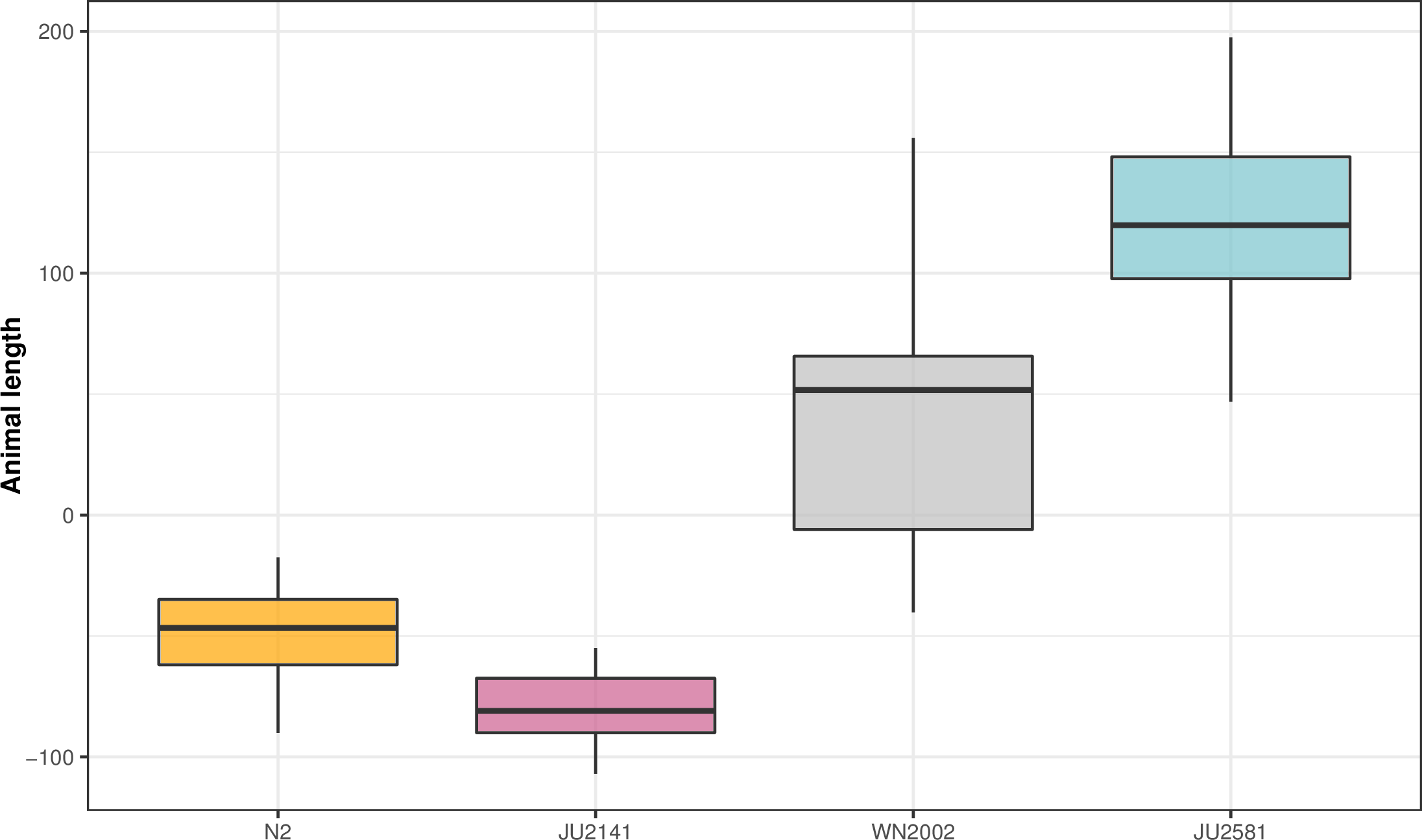

Supplement: S9 Fig — Tukey box plots of the animal length phenotypes of N2 and three wild isolates in the presence of ABZ. The y-axis represents the animal length phenotype after correcting for growth in DMSO conditions. (TIF) [file ppat.1007226.s034.tif]

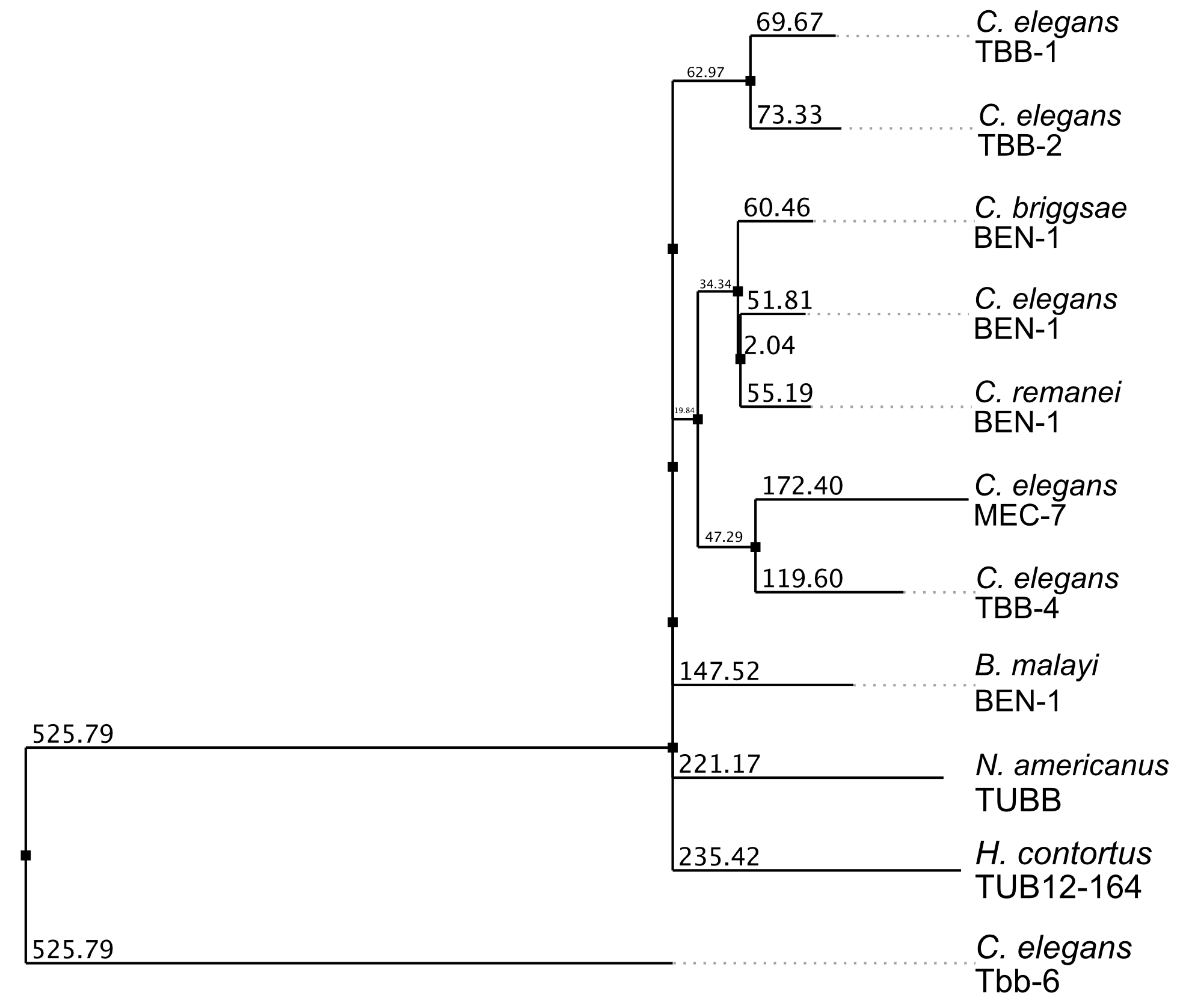

Supplement: S10 Fig — Neighbor-joining tree of the amino acid sequences of the six known C. elegans β-tubulins and paralogs in C. remanei, C. briggsae, H. contortus, and Necator americanus. (TIFF) [file ppat.1007226.s035.tiff]

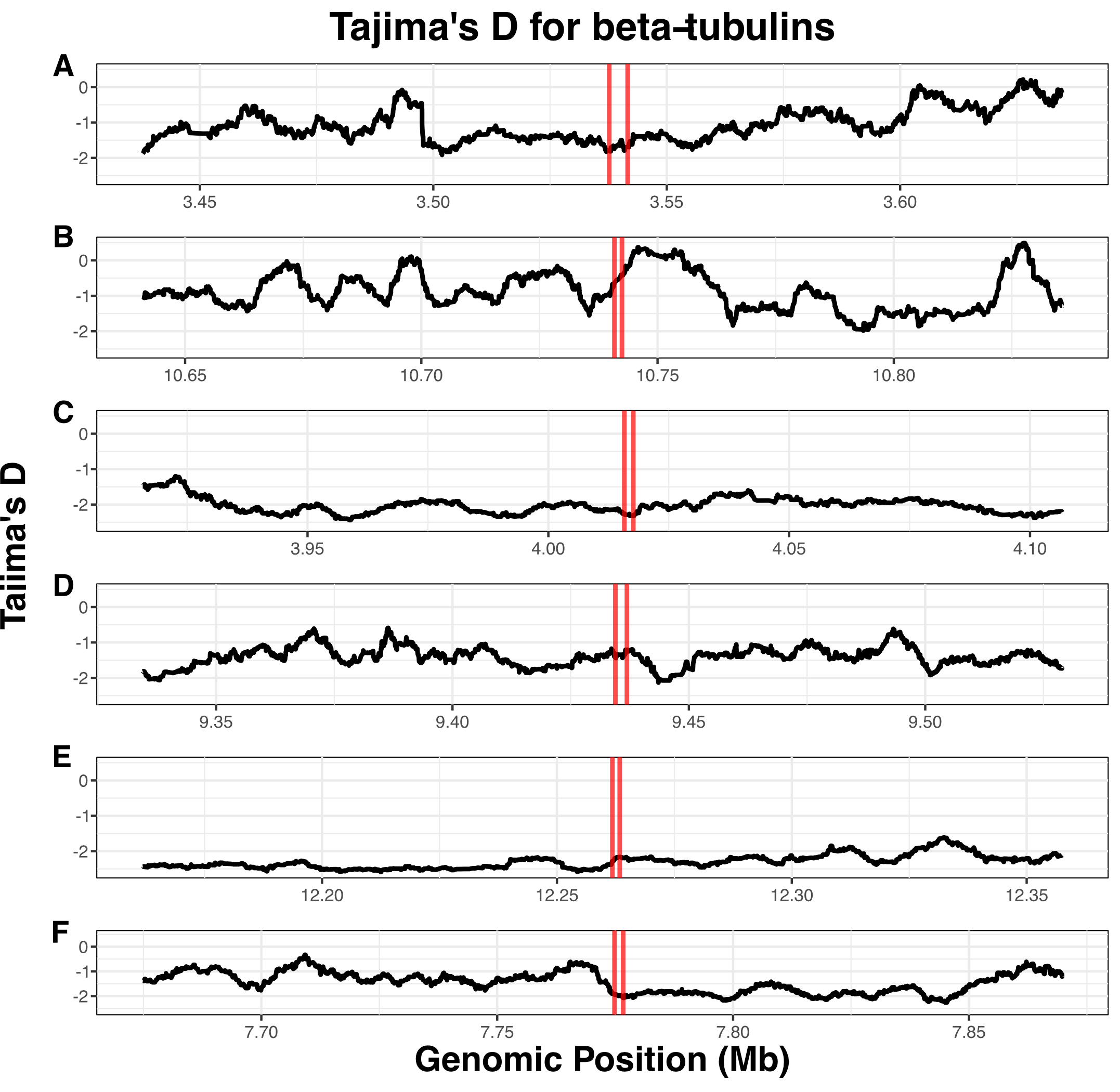

Supplement: S11 Fig — Tajima’s D calculated from SNV data of genomic regions surrounding (A) ben-1, (B) tbb-1, (C) tbb-2, (D) tbb-4, (E) tbb-6, and (F) mec-7. Genomic position in Mb is plotted on the x-axis, and Tajima’s D is plotted on the y-axis. Tajima’s D was calculated using a sliding window with a 100 SNV window and a one-SNV step size. Two red bars for each panel correspond to the start and end positions for the corresponding gene. (TIFF) [file ppat.1007226.s036.tiff]
